# Plant *in vitro* Culture Technologies; A Promise Into Factories of Secondary Metabolites Against COVID-19

**DOI:** 10.3389/fpls.2021.610194

**Published:** 2021-03-12

**Authors:** Tariq Khan, Mubarak Ali Khan, Kashmala Karam, Nazif Ullah, Zia-ur-Rehman Mashwani, Akhtar Nadhman

**Affiliations:** ^1^Department of Biotechnology, University of Malakand, Chakdara, Pakistan; ^2^Department of Biotechnology, Faculty of Chemical and Life Sciences, Abdul Wali Khan University Mardan (AWKUM), Mardan, Pakistan; ^3^Department of Botany, Pir Mehr Ali Shah Arid Agriculture University, Rawalpindi, Pakistan; ^4^Institute of Integrative Biosciences, CECOS University, Peshawar, Pakistan

**Keywords:** SARS-CoV-2, COVID-19, *in vitro* cultures, Plants-medicinal, biotechnology, secondary metabolites

## Abstract

The current pandemic has caused chaos throughout the world. While there are few vaccines available now, there is the need for better treatment alternatives in line with preventive measures against COVID-19. Along with synthetic chemical compounds, phytochemicals cannot be overlooked as candidates for drugs against severe respiratory coronavirus 2 (SARS-CoV-2). The important role of secondary metabolites or phytochemical compounds against coronaviruses has been confirmed by studies that reported the anti-coronavirus role of glycyrrhizin from the roots of *Glycyrrhiza glabra*. The study demonstrated that glycyrrhizin is a very promising phytochemical against SARS-CoV, which caused an outbreak in 2002–2003. Similarly, many phytochemical compounds (apigenin, betulonic acid, reserpine, emodin, etc.) were isolated from different plants such as *Isatis indigotica*, *Lindera aggregate*, and *Artemisia annua* and were employed against SARS-CoV. However, owing to the geographical and seasonal variation, the quality of standard medicinal compounds isolated from plants varies. Furthermore, many of the important medicinal plants are either threatened or on the verge of endangerment because of overharvesting for medicinal purposes. Therefore, plant biotechnology provides a better alternative in the form of *in vitro* culture technology, including plant cell cultures, adventitious roots cultures, and organ and tissue cultures. *In vitro* cultures can serve as factories of secondary metabolites/phytochemicals that can be produced in bulk and of uniform quality in the fight against COVID-19, once tested. Similarly, environmental and molecular manipulation of these *in vitro* cultures could provide engineered drug candidates for testing against COVID-19. The *in vitro* culture-based phytochemicals have an additional benefit of consistency in terms of yield as well as quality. Nonetheless, as the traditional plant-based compounds might prove toxic in some cases, engineered production of promising phytochemicals can bypass this barrier. Our article focuses on reviewing the potential of the different *in vitro plant* cultures to produce medicinally important secondary metabolites that could ultimately be helpful in the fight against COVID-19.

## Introduction

Severe acute respiratory syndrome coronavirus 2 (SARS-CoV-2) has become the deadliest virus in a century. SARS-CoV-2, which originated in Wuhan, China in December 2019, has killed more than two million people so far. This is the third time that a coronavirus has caused an outbreak during the 21st century, SARS and Middle East respiratory syndrome (MERS) being the previous ones. This virus has been termed the novel coronavirus (SARS-CoV-2) and causes a severe respiratory syndrome collectively called coronavirus disease 2019 (COVID-19). The disease, because of the ease of spread of its causative virus, became a pandemic very quickly. Owing to this, 2.37 million people have died because of COVID-19 and 108.33 million have tested positive for the virus as of February 13, 2021 (Worldometer, 2020).

Scientific advancements allowed researchers to advise governments across the world on quick prevention measures. Based on the rapid information coming out about the virus, including its transmission pattern, morphology, and deeper biological information, the World Health Organization (WHO) and other leading health organizations across the world advised on emergency containment and control measures. Control on a global scale allowed stakeholders from every sector to work toward mitigation measures more efficiently. Slowing down the spread and thus containment of the virus has also allowed scientists to work on many treatment options for COVID-19. Although the current option to treat COVID-19 patients is to alleviate symptoms and avoid co-infection with bacteria through medications, trials on different drug and vaccine candidates are underway (Thanh Le et al., 2020). However, the safety concerns regarding repurposed drugs and the fact that vaccines, when available, will only prevent infection, calls for additional avenues of drugs to treat patients. Plants provide one such avenue through the products of their secondary metabolism, i.e., phytochemicals. But these too are limited by safety concerns, seasonal and geographic dependence, and lesser uniformity in the metabolite profile of medicinal plants across the globe. The solution to these barriers in harnessing secondary metabolism running in plant cell factories is provided by plant biotechnology. Plant biotechnology is a very promising platform for providing uniform, safe to use, high-yield drugs against coronaviruses. This review article highlights the important potential role of plant cell factories to produce safe and high-yield medicinal compounds against COVID-19. The paper reviews the important biotechnological strategies that can be employed to make the best use of plants for providing secondary metabolites as candidates during anti-SARS-CoV-2 drug discovery.

## Treatment of COVID-19: A Brief Insight

Treatment options currently explored include passive immunity (Abraham, 2020), repurposing of existing drugs, and vaccine candidates (Harrison, 2020). For instance, recently, the already available dexamethasone, an inexpensive steroidal drug has been shown to save the lives of w-19 patients in a trial, called RECOVERY (Ledford, 2020). Similarly, chloroquine and hydroxychloroquine, antimalarial drugs also showed impressive results when repurposed to treat COVID-19 patients (Keyaerts et al., 2004; Gautret et al., 2020; Wang et al., 2020). Vaccine trials are also underway and the Moderna biotech vaccine candidate mRNA-1273 (approved for use now by the Food and Drug Administration) which encodes the stabilized pre-fusion SARS-CoV-2 spike protein has provoked an immune response with no trial-limiting side effects (Jackson et al., 2020). However, vaccines, based on their very mechanism of action, only prevent a healthy individual from getting infected. Moreover, a successful vaccine is not thought, at least soon, to be available to the masses. Similarly, repurposing synthetic drugs also became controversial because of their safety concerns and adverse events (Ferner and Aronson, 2020).

The fight against COVID-19 has now become one of the greatest challenges of the current times. The pandemic has lasted for over a year now since its inception in December 2019. To date, over 90 vaccines are being developed for the COVID-19 virus by different research groups in universities and major companies. Currently, two vaccines (Pfizer-BioNTech COVID-19 vaccine and Moderna COVID-19 vaccine) have obtained emergency use authorization from the Food and Drug Administration in the United States. Pfizer and Moderna have developed messenger RNA-based vaccines that have been shown to be 90–95% effective when given at preventing doses 21 and 28 days apart, respectively (Levenson and Howard, 2020). Some of the groups are even testing the direct injection of viral proteins that will help in eliciting the immune system and developing resistance against the virus. One fascinating approach is the use of genetically modified viruses to develop coronavirus proteins in the human body. The carrier virus will act as a vector, carrying coronavirus protein sequences in its genome. Currently, measles or adenovirus (where the viruses are weakened) is used in this approach to make either replicating or non-replicating virus versions (Callaway, 2020). Another fascinating alternative is the plant-based vaccines developed by Medicago (PMI, 2020). The company is developing a *Nicotiana benthamiana*-based virus-like particle (VLP), to develop a potential vaccine against the coronavirus disease. The VLPs use genetic sequencing from the coronavirus to mimic it and produce an immune response in the body. Plant-based VLP technology offers a very safe alternative to the vaccines already approved or in the process of development. These vaccines are virus-free and do not rely on animal products.

## Plant Secondary Metabolites and Their Antiviral Potential

Plant metabolism as a factory to produce anti-SARS-CoV phytochemicals is an important area of consideration currently. It is important to highlight the antiviral potential of the main classes of plant secondary metabolites to understand the role of *in vitro plant* cultures and associated biotechnological manipulation in fighting SARS-CoV-2. Plants produce a diversity of organic compounds classified as primary and secondary metabolites based on either being directly essential to the growth and development of plants (primary metabolites) or indirectly playing their role and not essential to growth (secondary metabolites). Secondary metabolites are produced in plants in situations of intracellular and/or extracellular stress and are used for interaction with the environment and protection from pathogens. This implies that there are thousands of secondary metabolites produced in plants, classified in different classes by their chemical structures. The four major classes of plant secondary metabolites are alkaloids, glycosides, phenolics, and terpenes. The purpose of highlighting the groups of these metabolites is to relate the role these classes of compounds could play against SARS-CoV-2. Several different plant-based compounds have been shown to be effective against the previous type of coronavirus, i.e., SARS-CoV. These compounds have been employed for their different mechanisms of actions against SARS-CoV (Table 1).

**TABLE 1:** Compounds active against SARS-CoV along with their reported anti-SARS-CoV mechanism of action.

	Compound	Plant	Virus acting on	IC_50_ value	Reported antiviral mechanism	References
1	Aescin	*Aesculus hippocastanum*	SARS-CoV	3.4 μmol/L	–	Wu et al., 2004
2	Celastrol	*Tripterygium regelii*	SARS-CoV	10.3 μmol/L	Inhibits SARS-CoV 3CLpro	Ryu et al., 2010b
3	Cepharanthine	*Stephania japonica*	SARS-CoV-2	0.98 μmol/L	ACE inhibitor	Fan et al., 2020
4	Chalcones I–IX	*Angelica keiskei*	SARS–CoV	11.4–129.8 μmol/L	Competitively inhibits SARS-CoV 3CLpro	Park et al., 2016
5	Dihydrotanshinone	*Salvia miltiorrhiza*	MERS-CoV	1 μg/mL	–	Kim et al., 2018
6	Emodin	*Rheum palmatum*	SARS-CoV	200 μmol/L	Blocks the binding of S protein to ACE2	Ho et al., 2007
7	Ginsenoside-Rb1	*Panax ginseng*	SARS-CoV	100 μmol/L	Inhibits glycoprotein activity	Wu et al., 2004
8	Glycyrrhizin	*Licorice root*	SARS-CoV	300 mg/L	Upregulates nitrous oxide synthase and nitrous oxide production	Cinatl et al., 2003; Schoeman and Fielding, 2019
9	Hirsutenone	*Alnus japonica*	SARS-CoV	4.1 μmol/L	Inhibits PLpro activity	Park et al., 2012a, b
10	Iguesterin	*Tripterygium regelii*	SARS-CoV	2.6 μmol/L	Inhibits SARS-CoV 3CLpro	Ryu et al., 2010b
11	Leptodactylone	*Boenninghausenia sessilicarpa*	SARS-CoV	100 μg/mL	–	Yang et al., 2007
12	Lycorine	*Lycoris radiata*	SARS-CoV	15.7 ± 1.2 nmol/L	–	Li et al., 2005
13	Myricetin	*Myrica rubra*	SARS-CoV	2.71 ± 0.19 μmol/L	Inhibits ATPase activity	Yu et al., 2012
14	Pristimererin	*Tripterygium regelii*	SARS-CoV	5.5 μmol/L	Inhibits SARS-CoV 3CLpro	Ryu et al., 2010b
15	Quercetin-3-β-galactoside	*Ginkgo biloba*	SARS-CoV	42.79 ± 4.97 μmol/L	Competitively inhibits SARS-CoV 3CLpro	Chen et al., 2006
16	Reserpine	*Rauvolfia serpentine*	SARS-CoV	6.0 μmol/L	–	Wu et al., 2004
17	Resveratrol	*Polygonum cuspidatum*	MERS-CoV	–	–	Lin et al., 2017
18	Saikosaponin B_2_	*Bupleurum chinense*	HCoV-229E	1.7 ± 0.1 μmol/L	Interferes with events of early viral attack	Li et al., 2005; Cheng et al., 2006
19	Scutellarein	*Scutellaria baicalensis*	SARS-CoV	0.86 ± 0.48 μmol/L	Inhibits ATPase activity	Yu et al., 2012
20	Tanshinones I–VII	*Salvia miltiorrhiza*	SARS–CoV	0.7–30 μmol/L	Inhibits PLpro activity	Park et al., 2012b
21	Tetrandrine	*Stephania tetrandra*	HCoV-OC43	0.33 ± 0.03 μmol/L	Inhibits p38 MAPK pathway	Kim et al., 2019
22	Theaflavin	Black tea	SARS-CoV-2	–	Inhibits RdRp activity	Lung et al., 2020

*SARS-CoV, severe acute respiratory syndrome-coronavirus; 3CLPro, 3C-like protease; PLpro, papain-like protease; MERS, Middle East respiratory syndrome coronavirus; ACE2, angiotensin-converting enzyme 2; H-CoV-229E, human coronavirus 229E; H-CoV-OC43, human coronavirus OC43; p38 MAPK, p38 mitogen-activated protein kinases; RdRp, RNA-dependent RNA polymerases.*

Alkaloids, for instance, are nitrogen-containing basic compounds and include compounds such as quinine, a bitter alkaloid isolated from the bark of the cinchona tree (Quina). A synthetic derivative of quinine, i.e., chloroquine has recently been tested and found to be a good drug candidate against SARS-CoV-2 because its DNA-intercalating properties prove potent in alleviating the symptoms of coronaviruses based on its biocompatibility (Devaux et al., 2020). Chloroquine, now being tested, has been found to result in side effects such as ventricular arrhythmias, serious cutaneous adverse reactions, and fulminant hepatic failure (Ferner and Aronson, 2020). Overall, despite the side effects, the experimentally proven efficacy of their analogs and derivatives mean that natural quinines could be effective in alleviating the symptoms based on their biocompatibility (Devaux et al., 2020). Similarly, reserpine, an alkaloid isolated from the dried root of *Rauvolfia serpentina* (Indian snakeroot) has been shown to inhibit the replication of SARS-CoV (the coronavirus that causes the first coronavirus-related epidemic of this century). Reserpine could thus prove to be an important candidate against SARS-CoV-2. Similarly, other important alkaloids, palmatine, and chelidonine were also reported as intercalating alkaloids and could be easily suggested as potential drug candidates against SARS-CoV-2 (Ho et al., 2007; Wink, 2020).

Similarly, flavone glycosides, phenolics, and polyphenolic compounds which are characterized by aromatic rings and hydroxyl (−OH) groups have also demonstrated important antiviral activity in many studies. For example, three flavone glycosides, quercetin 3-*O*-rutinoside, kaempferol 3-*O*-rutinoside, and kaempferol 3-*O*-robinobioside have proven effective against herpes simplex virus and thus points toward their potential role in working against human viruses (Yarmolinsky et al., 2012). It has been suggested that the −OH groups inhibit the activity of viral proteins by forming hydrogen bonds with the positively charged amino groups of proteins. Additionally, polyphenols can intervene in the lipoprotein layers of the viral envelope and thus prevent viral entry in the host cells (Wink, 2020). For instance, the flavonoid chrysin, derived from genus *Rheum* and *Polygonum* were tested positive for their achrysinctivity against the S protein and inhibition of ACE2 interaction (Ho et al., 2007). Flavonoids and polyphenolic compounds like luteolin and quercetin have experimentally proven activity against SARS-CoV. They have significantly blocked the entry of the virus into the cells. This was shown through studies of Yi et al. (2004) wherein they reported that these small molecules showed promising results with half-maximal effective concentration (EC_50_) of 83.4 and 10.6 μM, respectively.

Essential oils and terpenoids have an equally important role as antiviral plant secondary metabolites. Essential oils can enter non-specifically into the lipid bilayer of the viral envelope, altering the fluidity of the membrane and thus interfering with its pathogenicity even before the entry of the virus (Ben-Shabat et al., 2020). Terpenoids, comprised of isoprene units, terpenes, and their oxygenated derivatives, have also proved potent against many viruses including coronaviruses. For instance, α-cadinol, pinusolidic acid, and ferruginol, isolated from *Chamaecyparis obtuse*, betulonic acid, and cedrane-3β,12-diol, from *Juniperus formosana*, and cryptojaponol isolated from *Cryptomeria japonica* have been proven to be effective against SARS-CoV (Wen et al., 2007). The results of the study indicated that most of the terpenoids inhibited the replication of SARS-CoV at EC_50_ between 3.8 and 7.5 μM. Similarly, an important member of terpenoids, resveratrol has been shown to prevent the entry of MERS-CoV into the cell. Resveratrol fully prevented Vero E6 cell death at the concentration of 125–250 μM (Lin et al., 2017).

## Why Plant Biotechnology?

While the search for anti-SARS-CoV-2 drugs is ongoing, according to Capell et al. (2020), one avenue for looking for anti-SARS-CoV-2 drugs is the plant kingdom. In the traditional setup, raw plants, as well as extracts from plants, were used to treat different diseases. The WHO has suggested that 80% of the world’s population relies on plants for the treatment of many diseases (Bannerman et al., 1983). Plants have importantly been employed against human respiratory problems including respiratory viruses. Such is the importance of plant trials, that work is currently underway on dried fruit extracts of *Forsythiae fructus* as a part of the world’s search for an effective treatment for COVID-19 (Maxmen, 2020).

**FIGURE 1:**
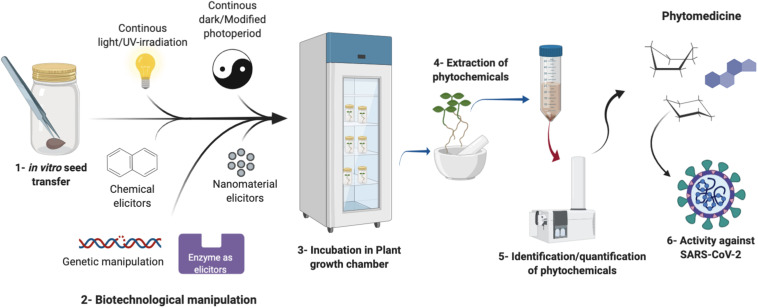
A schematic representation of the potential plant biotechnology methods that lead to the enhanced production of phytomedicine through *in vitro* cultures against SARS-CoV-2.

However, plants face the threat of over-harvesting and thus endangerment when collected rigorously. Similarly, due to insufficient data on safety-related aspects of the use of phytomedicine, concerns are still there. There is an incorrect perception that herbal drugs are fully safe and free from any side effects. There are hundreds of toxic constituents in different plants. For this purpose, detailed insight into the pathways and products of the plant’s secondary metabolism is important for drugs that are safe to use (Nature, 2020). Equally important is the fact that plants located in different regions of the world have different metabolite profiles and are highly dependent on geography and seasons. Plant biotechnology has the potential to overcome these barriers (Ramirez-Estrada et al., 2016). Plant *in vitro* cultures as an important pillar of plant biotechnology provides an option for making the best use of plant machinery to produce medicinally important secondary metabolites (Figure 1). Plant cell suspension cultures, callus cultures, hairy root cultures, adventitious root cultures, and other organ cultures can serve as the best sources of uniform production of phytomedicine for COVID-19 (Verpoorte et al., 2002). The importance of plant *in vitro* cultures lies in the reason that these cultures can be manipulated to trigger their defense response through activating their secondary metabolism. These triggers include elicitation by biotic and abiotic stresses given *in vitro* to produce enhanced quantities of phytochemicals. For instance, Ramirez-Estrada et al. (2016) reviewed the potential of methyl jasmonate as an important biotic elicitor to trigger the production of a diversity of secondary metabolites in different plant cell cultures (Ramirez-Estrada et al., 2016). Similarly, metabolic engineering backed by genetic manipulation tools has been a very viable biotechnology method to obtain novel metabolites and enhance the yield of the existing metabolites of interest (Gandhi et al., 2015).

### Micropropagation

Micropropagation is a robust and reliable technique used for the multiplication of plants through *in vitro* cultures; it produces many homogeneous plants in a short period. Besides, the production of bioactive secondary metabolites can be enhanced in medicinal plants with this technique (Khan I. et al., 2020). During micropropagation, tiny parts of the plants commonly called explants excised from different plant species can be micro-propagated under optimized growth conditions of culture media, temperature, and photoperiod (Abbasi et al., 2016).

As indicated in Table 2, several health-promoting metabolites, especially those which are reported for a multitude of antiviral potential, have been produced in many plants through micropropagation *in vitro*. For instance, Santoro et al. (2013) reported the production of higher quantities of pulegone and menthofuran in *Mentha piperita*, when micro-propagated *in vitro* under the effects of 4-indol-3-ylbutyric acid (IBA) and 6-benzylaminopurine (BAP).

**TABLE 2:** Production and enhancement of potential compounds against SARS-CoV-2 through plant biotechnological approaches.

Serial No.	Compound/class of compounds	Plant	*In vitro* culture type	*In vitro* culture conditions used	Plant growth regulator used	Elicitor used	Remarks (Results)	References
1	Saponins	Javanese ginseng (*Talinum paniculatum)*	Adventitious root cultures	Temperature 25 ± 1°C in the dark	Indole-3-butyric acid (IBA) or NAA (1-napthaleneacetic acid)	Methyl jasmonate (MeJA) and salicylic acid (SA)	1.5-fold upon elicitation with MeJA and 1.3-fold upon SA	Faizal and Sari, 2019
2	Astragaloside (AG)	*Astragalus membranaceus*	Hairy root cultures	Orbital shaker (100 rpm); 28 ± 1°C in the dark	–	Methyl jasmonate (MJ)	MJ-elicited (2.1- and 2.0-folds greater)	Jiao et al., 2016
3	Diosgenin	*Helicteres isora L.*	Callus and suspension cultures	Rotatory shaker (50–60) rpm; 25 ± 2°C temperature; 40 lmol m^–2^ s^–1^ light intensity; 16/8-h light/dark cycle	2,4-dichlorophenoxyacetic acid (2,4-D); kinetin (Kin); and 6-Benzylaminopurine (BAP)	*Escherichia coli*; *Bacillus subtilis*; *Saccharomyces cerevisiae*; and *Aspergillus niger*	E. coli (1.5%) proved best with a 9.1-fold increase	Shaikh et al., 2020
4	Gymnemic acid (GA)	*Gymnema sylvestre R. Br.*	Cell suspension cultures	Rotatory shaker (110 rpm); incubator at 25°C in dark; pH 5.8	2,4-dichlorophenoxyacetic acid (2,4-D); naphthaleneacetic acid (NAA); 6-benzyladenine (BA); picloram	Methyl jasmonate (MJ); yeast extract; chitin; and pectin	Yeast extract (5.25-folds); MJ (2.8-folds); pectin (2.65-folds); while chitin (2.62-folds)	Veerashree et al., 2012
5	Flavonoid	*Isatis tinctoria L.*	Hairy root cultures	Temperature 30°C; pH 7.0; and time 72 h	–	*Aspergillus niger* and *Aspergillus oryzae*	6.83-fold increase	Jiao et al., 2018
6	Rosmarinic acid	Purple basil (*Ocimum basilicum L. var. purpurascen*)	Callus cultures	Temperature (25 ± 2°C); pH of 5.6–5.7; 16/8 h light/dark	Naphthaleneacetic acid (NAA)	Melatonin; and UV-C irradiations	Melatonin (1.4-fold); UV-C radiations (2.3-fold) elevation	Nazir et al., 2020
7	Glycyrrhizin	*Glycyrrhiza glabra L.*	Hairy root cultures	Temperature 28 ± 2°C; 60 μ mol photon m^–2^ s^–1^ light for 16 h day and 8 h dark	Indole-3-acetic acid (IAA)	Abiotic elicitors: polyethylene glycol (PEG); CdCl2 Biotic elicitor: cellulase; mannan	PEG enhanced the yield up to 5.4-folds; cellulase (8.6-folds); Mannan (7.8-folds)	Srivastava et al., 2019
8	Chicoric acid	Purple basil (*Ocimum basilicum L. var. purpurascens*)	Callus cultures	Temperature (25 ± 2°C; pH of 5.6–5.7; 16/8 h light/dark	Naphthaleneacetic acid (NAA)	Melatonin and UV-C irradiations	Melatonin (3.2-folds) and UV-C radiations (4.1-folds)	Nazir et al., 2020
9	Quercetin	*Abutilon indicum L.*	Callus cultures	Temperature 25°C; pH 5.75 under dark conditions	2,4-dichloro phenoxy acetic acid (2,4-D) with indole-3-acetic acid (IAA)	phenylalanine (PA)	Three-fold increase	Sajjalaguddam and Paladugu, 2015
10	Peonidin	Purple basil (*Ocimum basilicum L. var. purpurascens*)	Callus cultures	Temperature 25 ± 2°C; pH of 5.6–5.7; 16/8 h light/dark	Naphthaleneacetic acid (NAA)	Melatonin and UV-C radiations	Melatonin (2.0-fold); and UV-C radiations (2.7-fold)	Jiao et al., 2018
11	Kaempferol	*Dysosma pleiantha (Hance) Woodson*	Callus cultures	Temperature 25 ± 1°C; pH 5.6–5.8 16 H photoperiod from white fluorescent lamps at a light intensity of 43 μmol m^–2^ S^–1^/8 h dark cycle	Medium (B5) 2,4-dichlorophenoxyacetic acid (2,4-D); kinetin	Casein hydro lysate; coconut water; and peptone extract	12.39-folds enhancement	Karuppaiya and Tsay, 2020
12	Ephedrine	*Ephedra alata*	Suspension cultures	Temperature 25 ± 2°C; pH 5.7–5.8; fluorescent light (2500–3000 lux); 16-h photoperiod	2,4-dichlorophenoxy acetic acid (2,4-D); and kinetin (Kn)	*Aspergillus niger* and yeast extract	Seven-fold increase	Hegazi et al., 2020
13	Caffeic acid	*Vitex agnus castus L.*	Agitated shoot cultures	Rotary shaker at 140 rpm	Naphthaleneacetic acid (NAA); benzyl aminopurine (BAP); gibberellic acid (GA3)	L-phenylalanine	1.5-folds increase	Skrzypczak- Pietraszek et al., 2018
14	Chlorogenic acid	*Cecropia obtusifolia*	Callus and cell suspension cultures	Rotatory shaker at 110 rpm; temperature 26°C; photoperiod of 16-h light with cool white fluorescent lamps at 50 lMm^–2^ s^–1^	Naphthalene acetic acid (NAA); 2,4-dichlorophenoxyacetic acid (2,4-D); indole-3-butyric acid (IBA); and indole-3-acetic acid (IAA); 6-benzylaminopurine (BAP)	Nitrate deficiency (lacking ammonium)	7.7-fold increase	Del Pilar Nicasio-Torres et al., 2012
15	Glycyrrhetinic acid	*Taverniera cuneifolia (Roth) Arn*	Root cultures	Microbial: 150 rpm; temperature 26 ± 2°C; for 24 h Methyl jasmonate (MJ): 120 rpm; 26 ± 2°C; 18/6 photoperiod white fluorescent light (30 μmol m^–2^ S^–1^)	–	Microbial elicitation (fungal and bacterial); methyl jasmonate (MJ)	Microbial elicitation (three-folds); methyl jasmonate (2.5) enhancement	Awad et al., 2014
16	Matairesinol	*Forsythia* × *intermedia*	Cell suspension cultures	Gyratory shaker at 110 rpm; 25°C; in the dark	–	Methyl jasmonate and coniferyl alcohol	Seven-fold increase	Schmitt and Petersen, 2002
17	Lignans	*Linum ussitatsimum L*	Cell suspension cultures	Gyratory shaker at 100 rpm placed in optimum conditions	Naphthalene acetic acid (NAA)	Salicylic acid (SA)	2.7-fold increase	Nadeem et al., 2019
18	Neochlorogenic acid	*Vitex agnus castus L.*	Agitated shoot cultures	Rotary shaker at 140 rpm	α-naphthaleneacetic acid (NAA); benzylaminopurine (BAP); gibberellic acid (GA3)	L-phenylalanine	16-fold	Skrzypczak- Pietraszek et al., 2018
19	Neolignans (dehydrodiconiferyl)	*Linum ussitatsimum L*	Cell suspension cultures	Gyratory shaker at 100 rpm with optimum growth conditions	Naphthalene acetic acid (NAA)	Salicylic acid	3.88-fold	Nadeem et al., 2019
20	p-coumaric acid	*Vitex agnus castus L.*	Agitated shoot cultures	Rotary shaker at 140 rpm	α-naphthaleneacetic acid (NAA); benzylaminopurine (BAP); gibberellic acid (GA3)	L-phenylalanine	5.3-fold	Skrzypczak- Pietraszek et al., 2018
21	(−) Menthone	Peppermint (*Mentha piperita*)	Micro propagation	Temperature 22 ± 2°C; pH 5.6–5.8; relative humidity (∼70%); light (16/8 h light/dark cycle)	Auxins 4-indole-3-ylbutyric acid (IBA); and the cytokinins 6-benzylaminopurine (BAP)	–	Two-fold increase	Santoro et al., 2013
22	Cynaroside	*Vitex agnus castus L.*	Agitated shoot cultures	Rotary shaker at 140 rpm	α-naphthaleneacetic acid (NAA); benzylaminopurine (BAP); gibberellic acid (GA3)	L-phenylalanine	1.5-fold	Skrzypczak- Pietraszek et al., 2018
23	(+)-Pulegone	Peppermint (*Mentha piperita*)	Micro propagation	Temperature 22 ± 2°C; pH 5.6–5.8; relative humidity (∼70%); light (16/8 h light/dark cycle)	Auxins 4-indole-3-ylbutyric acid (IBA); and the cytokinins 6-benzylaminopurine (BAP)	–	Three-fold increase	Santoro et al., 2013
24	Limonene	Pennyroyal (*Mentha pulegium)*	Cell suspension cultures	Shaker with 100 round per minute in 25 ± 1°C	2,4-D	Yeast extract; salicylic acid	Limonene increased with increasing concentrations of yeast extract elicitor	Darvishi et al., 2016
25	(+)-menthofuran	Peppermint (*Mentha piperita*)	Micro propagation	Temperature 22°C ± 2°C; pH 5.6–5.8; relative humidity (∼70%); light (16/8 h light/dark cycle)	Auxins 4-indole-3-ylbutyric acid (IBA); and the cytokinins 6-benzylaminopurine (BAP)	–	Two-fold enhancement	Santoro et al., 2013
26	Isoorientin (ISO)	*Cecropia obtusifolia*	Cell suspension cultures	Rotatory shaker at 110 rpm; temperature 26°C; photoperiod of 16-h light with cool white fluorescent lamps at 50 lMm^–2^ s^–1^	Naphthalene acetic acid (NAA); 2,4-dichlorophenoxyacetic acid (2,4-D); indole-3-butyric acid (IBA); indole-3-acetic acid (IAA); 6-benzylaminopurine (BAP)	Nitrate deficiency (lacking ammonium)	ISO synthesis was induced earlier and for longer time period	Del Pilar Nicasio-Torres et al., 2012
27	Gallic acid	*Barringtonia racemosa L.*	Cell suspension cultures	25 ± 2°C under 18 h light and 6 h dark	2,4-D and kinetin	Biotic (chitosan); abiotic (silver nitrate)	2.64-fold (silver nitrate); 1.34-fold (chitosan) increase	Osman et al., 2018
28	Aloe-Emodin	*Cassia tora*	Root cultures	Shaking at 60 rpm; 25 ± 2°C; in dark conditions	1-naphthaleneacetic acid and kinetin	Chitosan; yeast extract	Chitosan (8.82 times); yeast extract (6.21 times)	Teptat et al., 2020
29	Rosin	*Rhodiola rosea* (rose root)	Compact callus aggregate cultures	Shaken at 14.14 rad s^–1^ (135 rpm)	MS-Rh media supplemented with 6-benzylaminopurine (BAP); naphthalene acetic acid (NAA); sucrose	Cinnamyl alcohol	3 to 6-fold increase	György et al., 2004
30	Salidroside	*Rhodiola imbricata Edgew.*	Callus and suspension cultures	100 rpm shaker for 8 h and kept static for 16 h; pH 5.7	Indole-3-butyric acid (IBA); 6-Benzylaminopurine (BAP); gibberellic acid (GA3); kinetin (KN) and Thidiazuron (TDZ)	Chemical elicitors (growth hormones); physical elicitors (photosynthetic lights, ultraviolet light)	5.35-fold	Kad et al., 2018
31	Scopoletin	*Spilanthes acmella Murr.*	Cell Suspension cultures	Rotary shaker at 100 ± 10 rpm; 25 ± 2°C; 16–8 h light-dark regime, using fluorescent lamps at a light intensity of 35 μ mol m^2^ s^–1^	6-benzyladenine; 2,4-dichlorophenoxyacetic acid	Casein hydrolysate and L-phenylalanine	1.39-fold (casein hydrolysate); 3.43-fold (L-phenylalanine)	Abyari et al., 2016
32	Tyrosol	*Rhodiola crenulata*	Cell suspension cultures	Rotary shaker at 120 rpm; 25 ± 1°C; light intensity 24 lmo^*l*^/m^2^/s; 16 h light photoperiod	6-benzyaldenine (BA); naphthalene acetic acid (NAA) and thidiazuron (TDZ)	–	High level of tyrosol were detected	Shi et al., 2013
33	Wogonin	*Scutellaria lateriflora*	Hairy root cultures	Shaking (121 rpm) at 26 ± 1°C	Phytohormone-free MS medium having sucrose and supplemented with antibiotic ampicillin and cefotaxim	*YE and bacterial suspensions (A) A. rhizogenes A4, (B) Pectobacterium carotovorum 1043 (Pba 1043), (C) Pseudomonas syringae var. syringae 764 (Pss 764), (D) Klebsiella pneumoniae 3896, and (E) Enterobacter sakazakii*	4.4-fold increase	Wilczañska-Barska et al., 2012
34	Rutin	*Vitex agnus castus L.*	Agitated shoot cultures	Rotary shaker at 140 rpm	α-naphthaleneacetic acid (NAA); benzylaminopurine (BAP); gibberellic acid (GA3)	L-phenylalanine	2.8-fold enhancement	Skrzypczak- Pietraszek et al., 2018
35	Anthocyanin	Purple basil (*Ocimum basilicum L. var. purpurascens)*	Callus cultures	Temperature 25 ± 2°C; pH 5.6–5.7; 16/8 h light/dark	NAA (2.5 mg/L)	Melatonin and UV-C radiations	Melatonin (3.7-fold) and UV-C radiations (4.1-fold) increase	Nazir et al., 2020
36	Cynaroside	*Vitex agnus castus L.*	Agitated shoot cultures	Rotary shaker at 140 rpm	α-naphthaleneacetic acid (NAA); benzylaminopurine (BAP); gibberellic acid (GA3)	L-phenylalanine	1.5-fold yield increase	Skrzypczak- Pietraszek et al., 2018
37	Luteolin	*Dracocephalum kotschyi* Boiss.	Seed germination	Temperature 28°C day/20°C night; 50% air relative humidity; photoperiod of 16 h light and 8 h dark	Melatonin (N-acetyl-5-methoxytryptamine); Calcium (Ca^2+^)	Salinity stress	Salinity stress alone (3.21-fold); salinity stress with melatonin and Ca^2+^ (2.83-fold increase)	Vafadar et al., 2020
38	Saikosaponins	*Bupleurum falcatum L.*	Root cultures	Gyratory shaker 105 rpm; 23 ± 2°C; 12:12 light-dark cycle (h)	Indole-3-butyric acid (IBA)	Two step sucrose concentration	Four-fold yield increase	Kusakari et al., 2000
39	Phenolic compounds	*Morinda coreia* Buck. And Ham.	Adventitious roots cultures	Agitated in dark on gyratory shaker at 100 rpm; 25 ± 2°C temperature for 8 days	Indole-3-butyric acid (IBA); BAP and Kin	Chitosan	1.21-fold more than IBA treated root suspension culture	Kannan et al., 2020
40	Essential oils (Thymol and p-cymene)	*Carum copticum L.*	Callus cultures	Temperature 25°C; pH 5.8; 16-h photoperiod supplied by white fluorescent lamps at 90 lmol m^–2^ s^–1^ in growth chamber	2,4-dichlorophenoxyacetic acid (2,4-D); benzyl amino purine (BAP)	Salt stress and chitosan	Thymol (from 14.5 to 25.1-fold); p-cymene (from 10 to 14.5-fold increase)	Razavizadeh et al., 2020

*CdCl, cadmium chloride; MSRs, Murashige and skoog-Rhodiola rosea medium; IBA, indole-3-butyric acid; NAA, naphthaleneacetic acid; MeJa, methyl jasmonate; SA, salicylic acid; 2, 4D, 2,4-dichlorophenoxyacetic acid; Kn, kinetin; BAP, 6-Benzylaminopurine; PEG, polyethylene glycol; GA, gibberellic acid; TDZ, thidiazuron; BA, 6-benzyaldenine.*

**FIGURE 2:**
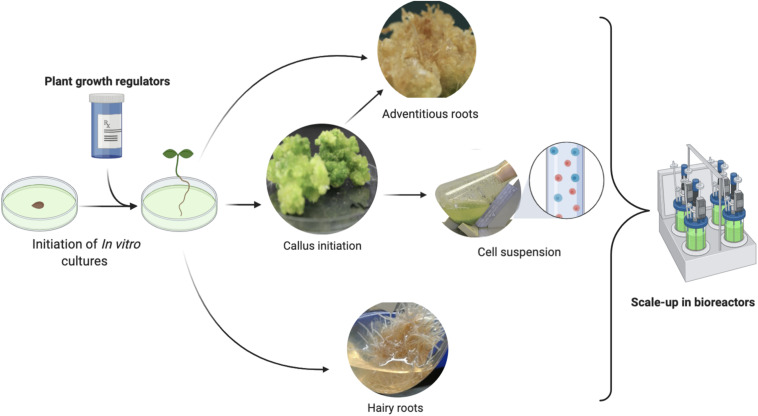
A pictorial representation of the various *in vitro* cultures generated through plant biotechnology methods and their potential for the commercial scale production of phytomedicine.

In a recent study, Ali et al. (2018b) reported that *Ajuga bracteosa* (a high-valued medicinal plant) accumulated higher levels of monoterpene hydrocarbons, which could be potentially used as essential oil-based medicine against human viruses. These hydrocarbons included limonene (3.4%), α-pinene (5.3%), camphene (4.45%), α-thujone (9.4%), 1,8-cineole (14.3%), borneol (11.4%), camphor (12.2%), and nerol (9.2) in the shoots raised *in vitro* in response to the application of TDZ (Ali et al., 2018a). Similarly, the supplementation of TDZ into the MS media produced a substantial amount of monoterpenes and sesquiterpenes through shoot cultures in the medicinally potent plant *Lallemantia Iberica* (Pourebad et al., 2015). The higher production of the important terpene volatiles (candidate anti-SARS-CoV-2 metabolites) in the regenerated shoots can be attributed to the different attributes of shoot cultures, such as the juvenile stage of the differentiated shoots, as the monoterpenes biosynthesis is directly linked to the young and immature shoot with higher metabolic potential (Bassolino et al., 2015). Biosynthesis of terpene metabolites generally takes place in epidermal cells of shoot or leaf and is stored in special glandular structures called leaf trichomes (Ali et al., 2018a). In another study, compared with callus cultures, the *in vitro* raised shoot cultures in medicinally important plants *Lavandula angustifolia* and *Rosmarinus officinalis* were found to accumulate higher levels of monoterpenes hydrocarbons (Gounaris, 2010). As the growth and development during *in vitro* shoot cultures are highly influenced by the effects of different plant growth regulators, the biosynthesis of terpenes could be correlated to *in vitro* growth and development. The ontogenetic changes in the shoots as a result of plant cell growth and the accelerated but controlled secondary metabolism during *in vitro* cultures are other important reasons which influence and regulate the biosynthesis of secondary metabolites (Khan et al., 2019a, b; Khan M. A. et al., 2020). Apart from micro-propagated plantlets many other *in vitro* cultures are also serving as useful sources of different medicinally important secondary metabolites (Figure 2).

### Callus and Cell Cultures

Plant cell cultures compared with wild plants and other types of cultures have the advantage of being (1) less prone to various environmental variations, (2) stable production platforms of homogeneous and uniform yield, (3) rapid growth, (4) reproducible, and (5) able to synthesize novel products that do not normally exist in the native plants (Khan et al., 2017; Khan M. A. et al., 2020). In addition to medicinal products, cell suspensions have been employed to produce compounds used as fragrances, food flavors, and additives, dyes, and coloring agents (Saeed et al., 2017). A lot of important medicinal plants have been exploited for the production of useful antiviral medicinal metabolites through callus and cell cultures (Ali et al., 2018a, b). For example, as listed in Table 2, considerable levels of diosgenin (an anti-SARS-CoV metabolite) were detected in the callus cultures of *Helicteres isora* L (Shaikh et al., 2020). Callus and cell cultures in *Gymnema sylvestre* have shown an optimal production of gymnemic acid (GA) which possesses the potential to work against SARS-CoV-2 (Veerashree et al., 2012). In some studies, the cell cultures were found to only accumulate the precursors of volatile medicinal compounds; while, cultures of other plants such as mentha have been recommended to produce high-valued medicinal monoterpenes compared to those found in the intact mentha plants. Likewise, callus cultures of *M. piperita* have been reported for the accumulation of monoterpenes in special secretory organs (Khan et al., 2019a). Similarly, results from another study have shown that callus cultures of *Carum copticum* also accumulated higher levels of thymol and p-cymene than their wild-grown respective plants (Razavizadeh et al., 2020).

Light intensity or quality during *in vitro* cultures can influence the plant cell’s physiological and hormonal status through the initiation of distinct metabolic pathways that ultimately influence and regulate the biosynthesis of important essential oils (Giri and Zaheer, 2016; Ali et al., 2018b). In cell cultures of *Ocimum basilicum*, constant light illumination produced higher total essential oil yield including the potent volatile linalool than the cell cultures grown under complete darkness. The process of elicitation by application of chemical elicitors, e.g., phenylacetic acid and methyl jasmonate and under the effects of physical elicitors such as the absence of light illuminance in the cultures have positively influenced the production of medicinally potent metabolites in an *A. bracteosa* cell culture (Ali et al., 2018b). In another study, important monoterpenes such as limonene and terpinolene (potential anti-SARS-CoV-2 metabolites) were elicited by methyl jasmonate under dark in higher amounts in *Rosa damascene* cell cultures (Olgunsoy et al., 2017). The process of elicitation is directly linked with the biosynthesis of essential oils in the plant cell. Several factors are responsible for the regulation of volatile compound biosynthesis. These factors include the genetic makeup of the explant used in cell cultures, the type of culture media, and the *in vitro* developmental phase of plant cells (Holopainen, 2011). There are, however, many limitations to cell suspension culture technology including slow growth and scale-up hurdles, the instability of cell lines, and subsequent lower yield of some important metabolites (Abbasi et al., 2016). Nevertheless, optimization of cell cultures could result in the generation of very viable factories to produce medicinal compounds that could work against SARS-CoV-2 and other human viruses.

### Hairy Roots

Generally, the potential of plant cell cultures to produce bioactive secondary metabolites can be enhanced by the induction of cell differentiation. Within the different cell culture approaches, hairy root cultures hold tremendous potential for the biosynthesis of volatile organic compounds besides other classes of potent secondary metabolites. When plant tissue is genetically transformed by *Agrobacterium rhizogenes* which inserts its T-DNA through the Ri plasmid, it results in the formation of hair-like small and fine roots. The advantage of hairy root culture technology is that it does not require further media supplementation with plant growth regulators because the inserted T-DNA carries the genes responsible for the endogenous biosynthesis of auxins. Lacking the property of geotropism, hairy roots are highly branched and can grow faster than normal roots. They not only produce the metabolites at levels like the normal roots but also generate metabolites that are produced in the aerial parts of the natural plants. Furthermore, hairy roots are physiologically and biochemically stable like any other cell culture technology (Gounaris, 2010). An excellent study has concluded the potential of hairy roots culture technology for the optimal production of antiviral flavonoids in *Isatis tinctoria* (Jiao et al., 2018). *Isatis tinctoria* (A.K.A. *Isatis indigotica*) has been shown to possess potential against SARS-CoV through its root-derived phytochemicals (Li et al., 2005). Among the different plants, the hairy roots of *Pimpinella anisum* and *Achillea millefolium* resulted in the production of medicinally important essential oils (Santos et al., 1998). In certain cases, such as the hairy roots of *Daucus carota* and *Laburnum alpinum*, the essential oil profiles of the volatiles were found in elevated levels, compared with the respective callus and cell cultures. Further, the metabolic pathways for the biosynthesis of volatiles can be manipulated by using more effective transgenes that can be inserted into the T-DNA region.

## Elicitation of *in vitro* Cultures; a Promising Avenue for Anti-Coronavirus Medicinal Compounds

Apart from the diversity of *in vitro* cultures, which can provide avenues for phytochemical compounds against SARS-CoV-2, enhanced production of these compounds is one area where plant biotechnology thrives. This enhancement is achieved through triggering or in other words eliciting the defense response of plant cultures, discussed in the previous section. To give a very brief overview of the mechanism of elicitation, the process starts at the cell membrane of the plant cell. Many different receptors are elicited to trigger the secondary metabolism for defense. For instance, the plasmalemma membrane-associated receptors attach the ligand or chemical compound (exogenous or endogenous). The signal reception is followed by transduction which includes steps like reversible phosphorylation and dephosphorylation of plasma membrane and cytosolic proteins, enhancement of Ca^2+^ in the cytosol, H^+^ influx/Cl^–^ and K^+^ efflux, extracellular alkalinization and cytoplasmic acidification, mitogen-activated protein kinase (MAPK) activation, NADPH oxidase activation and production of reactive oxygen and nitrogen species (ROS and RNS), early defense gene expression, jasmonate production, late defense response gene expression, and secondary metabolite accumulation (Ramirez-Estrada et al., 2016).

Being of biological origin (biotic) or non-biological origin (abiotic), the compounds or physical factors that stimulate the plant defense are termed elicitors. Biotic elicitors include compounds that are of the pathogenic origin or are produced by the plants after being exposed to pathogens. Abiotic elicitors include chemical compounds such as salts or physical factors such as environmental triggers (Devaux et al., 2020). The most relevant example of elicitation of important anti-SARS-CoV metabolites is that of glycyrrhizin from *Glycyrrhiza glabra* L. *G. glabra* L. has become an endangered medicinal plant due to the unabated extraction of glycyrrhizin (Srivastava et al., 2019). Glycyrrhizin is a triterpenoid saponin and has been shown to possess strong antiviral activity in killing SARS-CoV in a lancet study (Cinatl et al., 2003). Srivastava et al. (2019) have successfully elicited the yield of glycyrrhizin in hairy root cultures of *G. glabra* L. Through this study, it was proven that both biotic and abiotic elicitors are effective in eliciting higher yields of glycyrrhizin.

### Biotic Elicitors Can Trigger the Production of Plant Secondary Metabolites Against SARS-CoV-2

Compounds of a biological origin that elicit plant defense response and thus produce higher quantities of secondary metabolites are produced in two ways. Biotic elicitors are either compounds coming from pathogens, i.e., exogenous in origin or are compounds/hormones produced as a response to the pathogen that in turn triggers the plant’s defense response (endogenous elicitors). Plant *in vitro* cultures have been used as factories for the enhanced production of medicinally secondary metabolites through the application of many different exogenous and endogenous elicitors (Srivastava et al., 2019). Exogenous biotic elicitors include bacterial lysates, microbial enzymes, polysaccharides (chitin), and yeast extracts. For instance, cellulase, which directly serves bacteria and fungi and helps in attacking plant cell walls, has been shown to enhance the production of glycyrrhizin up to 8.6-fold through the application of cellulase to the hairy roots of *G. glabra* L. Besides, mannan oligosaccharides derived from the cell wall of yeasts (*Saccharomyces cerevisiae*) (De Oliveira et al., 2014) have also been reported to trigger the enhanced production of glycyrrhizin (7.8-fold compared to control) in hairy root cultures of *G. glabra* L. It should be reiterated here that glycyrrhizin possesses a demonstrated activity against the previously epidemic SARS-CoV. This saponin from licorice roots can inhibit the replication of SARS-associated coronavirus with an EC_50_ value ranging from 300 to 600 mg/L (Cinatl et al., 2003). Similarly, methyl jasmonate, a very important endogenous biotic elicitor has been shown to be effective in enhancing the production of glycyrrhizin up to almost 109 micrograms/g dry weight after 5 days of elicitation with 100 mM of methyl jasmonate. The study also demonstrated the role of other elicitors such as chitosan and yeast extract on the production of glycyrrhizin and demonstrated their effectiveness (Wongwicha et al., 2011). Other biotic elicitors ascorbic acid, eugenol, salicylic acid, and yeast extract have been employed for the enhancement of glycyrrhizin in cell cultures of *Abrus precatorius* (Karwasara et al., 2010).

Similarly, a higher yield of *trans*-resveratrol, previously shown to act strongly against MERS-CoV, has been reported in cell suspension cultures of *Vitis vinifera* through the application of 2, 3-dihydroxypropyl jasmonate (Shen et al., 2012). Chitosan is a polysaccharide that acts as a biotic elicitor and is used for high-yield production of medicinally important secondary metabolites (Hadwiger, 2013). Results from the study of Ferri et al. (2009) revealed that chitosan enhanced the production of important polyphenols, including stilbenes and flavonoids in cell cultures of *V. vinifera*. There are innumerable studies available on enhancing the yields of many important plant secondary metabolites that could be very effective in dealing with SARS-CoV-2. Plant secondary metabolites such as lycorine, reserpine, plant lectins, apigenin, luteolin, and quercetin (replication inhibitors of coronavirus) (Wu et al., 2004; Li et al., 2005; Keyaerts et al., 2007; Ryu et al., 2010a) have been elicited through the use of biotic elicitors including methyl jasmonate, salicylic acid, and chitosan (Dyakov et al., 2007; Ptak et al., 2017; Chandran et al., 2020).

### Enhanced Production of Anti-coronavirus Plant Secondary Metabolites Through Abiotic Elicitors

Just like biotic elicitors, chemical compounds of abiotic origin and physical factors such as environmental stimuli have also been proven effective in the elicitation of plant defense and thus increased production of phytochemicals (Halder et al., 2019). It is not possible to cover all abiotic elicitors in a single subsection, but for our purpose, abiotic elicitors compounds such as salts (e.g., AgNO_3_, CdCl_2_, etc.) and environmental stimuli such as continuous light/dark, different wavelengths of light, and osmotic stress, etc. have been employed to produce high-yield secondary metabolites in plant *in vitro* cultures.

Abiotic elicitation has been used for the enhanced production of important flavonoids such as hypericin and hyperforin in *in vitro* cultures of *Hypericum perforatum* (Shakya et al., 2019). For instance, Tirillini et al. (2006) reported that the application of chromium (0.01 mM) increased the production of hypericin by 38% in plantlets of *H. perforatum* (Tirillini et al., 2006). Compounds such as quercetin in *H. perforatum* have been shown to act as potent anti-SARS-CoV compounds and their enhanced production through elicitation promises an avenue for high-yield drug production (Ryu et al., 2010a; Shakya et al., 2019). A new addition to the lines of abiotic elicitors is the use of nanomaterials for triggering an intense plant defense response. For example, in one of our studies, we used silver nanoparticles (AgNPs) for the enhancement of medicinally important phenolics and flavonoids in callus cultures (Begum et al., 2020). Similarly, zinc nano-oxides and iron nano-oxides have been used to trigger the production of hypericin and hyperforin in cell suspension cultures of *H. perforatum* (Shakya et al., 2019). Apart from chemical compounds, environmental triggers have also proved to be valuable tools in plant biotechnology for the enhanced production of important secondary metabolites in plant *in vitro* cultures. For example, Khan et al. (2019c) showed that different spectral lights result in the enhanced production of phytochemicals such as myricetin and apigenin among many others. Myricetin is experimentally shown to inhibit the SARS-CoV helicase protein *in vitro* by affecting ATPase activity and thus could have potential against SARS-CoV-2 (Yu et al., 2012). Plants *in vitro* cultures can serve as factories for the elicitor-induced high-yield production of myricetin against SARS-CoV-2. In another study, Huang et al. (2016) used UV-B irradiation to cause flavonoid-related gene expression in hairy root cultures of *Fagopyrum tataricum*. The experiment resulted in enhanced biosynthesis of rutin and quercetin in the hairy root cultures of *F. tataricum*.

Conclusively, the application of biotic and abiotic elicitors during plant *in vitro* cultures is a promising avenue for the production of enhanced quantities of drug candidates against SARS-CoV-2.

## Genetic Engineering of Plants for Enhanced Metabolites Biosynthesis

Few reports are available on the genetic engineering of different plant species through a transformation with the candidate genes responsible for medicinal metabolites biosynthesis. Particularly, the metabolic pathways responsible for producing antiviral metabolites. In these studies, the cauliflower mosaic virus *promoter* (*CaMV 35S*) was used for the overexpression of the reductoisomerase DXR of the mevalonate MEP pathway in peppermint, and a significantly higher (50%) increase in total essential oil production was observed. The yields of cyclic monoterpenes were enhanced by the overexpression of the limonene synthase enzyme in the plastid. The overexpression of the rate-limiting factors significantly enhanced the specific yield of monoterpenes (Daviet and Schalk, 2010). It is crucial in some instances to enhance the yield of specific compounds of interest such as the monoterpenes α-pinene and d-limonene which are suitable alternatives to hazardous chemicals. Thus, the *in vitro* cultures through a genetic transformation in plants can boost the production of the desired compounds (Roberts, 2007). For instance, the production of monoterpene alcohols can be accelerated by the overexpression of linalool synthase, the enzyme responsible for the profound production of glycosylated forms than the free form. Likewise, overexpression of prenyltransferase has been found to increase the yields of the linear as well as some cyclic sesquiterpenes (Gounaris, 2010).

## Metabolic Engineering of Plants for Production and Enhancement of Anti-Coronavirus Compounds

**FIGURE 3:**
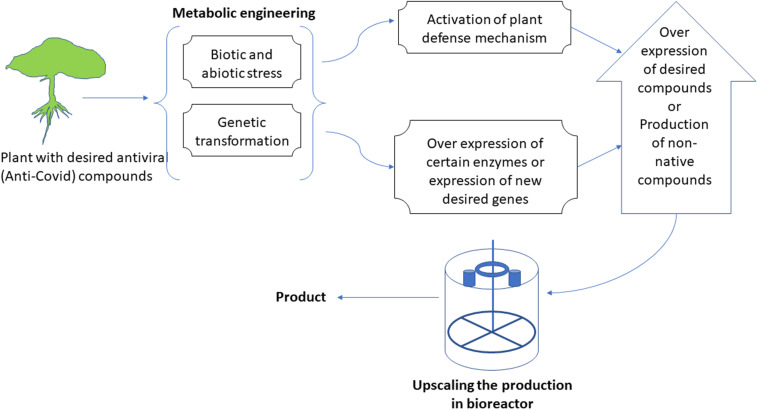
A representative flow-chart for the process of metabolic engineering of in vitro cultures to produce novel compounds or to over-produce existing compounds.

The metabolic machinery of plants could be targeted for engineering at different points that ultimately result in either novel compounds or the over-production of important medicinal metabolites (Figure 3). The metabolic engineering of plants is one area of plant biotechnology that possesses enormous potential for producing anti-SARS-CoV-2 compounds. Many of such pharmacological compounds have been extensively studied and reported in the literature (De Luca et al., 2012; Atanasov et al., 2015; Wurtzel and Kutchan, 2016; Buyel, 2018). Traditionally, people take these compounds orally as a whole plant, its decoction, or as a crude extract. However, it can be detrimental as most of the time unwanted compounds are also administered. Besides, these compounds are present at low native concentrations in plants and most of the time is not as effective as a pure compound. Commercial extraction of any such compound from any plant species may have detrimental effects on the plant population and can even push a plant species to the brink of extinction (Barone et al., 2019). For example, mass production of paclitaxel (source of Taxol^®^) led to the endangerment of not only its source plant (Pacific yew) but also other species of the same genus (Hawkins, 2007).

Thus, an increasing interest has been observed focusing on *in-planta* production of important metabolites via genetic and metabolic engineering. For example, genistein and taxane (precursors of paclitaxel) have been successfully produced in plants other than their native species. However, alongside attempting the increased production of certain end-products or producing new products/compounds via genetic engineering, it has been understood that biosynthetic engineering is a highly complicated process that demands diverse knowledge in all fields of biochemistry, biotechnology, and molecular biology (Barone et al., 2019). Several *in vitro* cultures for growing plant tissues could be manipulated through metabolic engineering for secondary metabolite production and its quantity enhancement, for example; adventitious root culture, callus culture, somatic embryogenesis and regeneration, cell suspension culture, protoplast culture, and hairy root culture, etc. (Gantait et al., 2020).

Despite drawbacks, attempts to overproduce a single metabolite or end-product have progressed in recent years and several examples can be found in the literature in which metabolic engineering of plants has been applied in the field of medicine. For instance, the production of genistein, an isoflavone, and a known antiviral compound has been shown in non-leguminous species in which this compound is not native. It was made possible by introducing the isoflavone synthase (IFS) gene from soybean (glycine max) to non-leguminous species tobacco (*Nicotiana tabacum*), lettuce (*Lactuca sativa*), and petunia (*Petunia hybrida*) (Barone et al., 2019). Furthermore, to increase the quantity of genistein, anti-sense suppression of flavanone-3-hydroxylase (F3H) and overexpression of phenylalanine ammonia-lyase (PAL) has also been employed. It is important to highlight here that PAL is an enzyme of the phenylpropanoid pathway that feeds into flavonoid biosynthesis.

Artemisinin commercially known for its antimalarial activity is also reported for its antiviral activity (Lubbe et al., 2012). The low concentration of artemisinin in its native plant the sweet wormwood (*Artemisia annua*) and its high demand in the pharmaceutical industry have led researchers to investigate its *in-planta* production as well as in other culture systems. Although little success has been made in this direction, a potential bottleneck has been identified which may lead to its biosynthesis in the near future.

In another study, sweet wormwood has been successfully transformed using *Agrobacterium tumefaciens* to produce taxane (a paclitaxel precursor) (Li et al., 2015). It should be noted that paclitaxel is a famous anticancer compound and is also known for its anti-HIV activity (Ryang et al., 2019).

Glycyrrhizin, an active component of licorice roots, has been reported to show antiviral activity against SARS-CoV *in vitro* (Boccalone et al., 2020). This compound has been converted via biotransformation to several other compounds that are more stable, easily soluble, and having greater emulsifying properties than glycyrrhizin. Other advantages of biotransformation of glycyrrhizin include strong stereoselectivity and regioselectivity, low byproduct production, and increased activity (He et al., 2019). It has been found that cell suspension culture of *G. glabra* and *Eucalyptus perriniana* can transform glycyrrhetinic acid (a byproduct of glycyrrhizin) to several other important compounds. For instance, these include 3−*O*−[α−l−arabinopyranosyl−(1→2)−β−D−glucuronopyran osyl]−24−hydroxy−18 β−glycyrrhetinic acid, 23,28−dihydroxy−18β−glycyrrhetinic acid−30β−glucopy ranosyl ester, and 28−hydroxy−18β−glycyrrhetinic acid−30β−glucopyranosyl ester which are reported to be important medicinal compounds (Hayashi et al., 1990; Orihara and Furuya, 1990).

Resveratrol is another example of a natural product that is reported for its anti-coronavirus activity (Lin et al., 2017) and has been successfully transformed via molecular engineering into plants that normally do not produce resveratrol (Giovinazzo et al., 2012). This compound has been reported to be found in grapes, berries, white tea, and passion fruit, etc. in a very low quantity which makes its extraction challenging (Shrestha et al., 2019). Metabolic engineering has been performed by several researchers to improve its quantity or to express it in new plants (Giovinazzo et al., 2012). For instance, the expression of grape genetic markers in tobacco leaves diverted the typical substrates of chalcone synthase to produce CHS up to 300 mg/g of resveratrol (Halls and Yu, 2008).

Plant metabolic engineering could prove to be an important tool in directing a plant’s metabolic machinery to the synthesis of important natural compounds against coronaviruses. For instance, metabolically engineered soybean and canola produced a high level of monosaturated fatty acid which otherwise produces a high level of linolenic acid which is prone to oxidation (Dellapenna, 2001). In another example, the use of g-tocopherol methyltransferase (g-TMT) showed a 10-fold increase in vitamin E activity in engineered Arabidopsis seed oil (Shintani and Dellapenna, 1998). In an example of metabolic engineering, b-carotene (provitamin A) has been successfully engineered into rice endosperm (Ye et al., 2000). 3-*O*-glucosyl-resveratrol production in a *V. vinifera* cell culture was significantly stimulated by saliva, with a 7.0-fold increase compared to control (Cai et al., 2012). Methyl jasmonate elicitation is an effective strategy to induce and enhance the synthesis of the anticancer agent paclitaxel (Taxol^®^) in taxus cell suspension cultures (Patil et al., 2014).

In an interesting experiment, *N. tabacum* plants were transformed with the stilbene synthase gene from grapevine using *A. tumefaciens*-mediated gene transfer. The transformed plants not only expressed the gene but also showed resistance to the fungal pathogen *Botrytis cinerea* (Hain et al., 1993). Metabolic engineering has thus improved its composition as well as improved its level. Stilbene synthase gene (STS-encoding gene)-mediated transformation thus confirmed that plant molecular engineering with resveratrol may lead to novel functions such as resistance to stresses, fungal infection, or increased nutritional value. STS genes have been transferred to several other crops as well such as *Medicago sativa L.*, *Arabidopsis thaliana L*, *L. sativa L*, and *Solanum lycopersicum L.*, etc. (Giovinazzo et al., 2012). The gene expression is however controlled by the chosen promoter. Commonly used promoters to control the expression of STS-encoding genes include the well-characterized constitutive promoter pCaMV35S, fungus-inducible promoter pPR10.1, stress-responsive promoter pVst1, and the tissue-specific promoter p-nap (Stark-Lorenzen et al., 1997; Coutos-Thévenot et al., 2001; Fan et al., 2008). The increased nutritional values of several fruits and edible crops via transformation along with the higher yield of resveratrol can be exploited for use in SARS-CoV-2 management.

Ginsenoside (ginsenoside Rb1) occupies a unique place in the pharmaceutical industry around the globe as an active ingredient of *Panax ginseng*. It has recently been reported for its anti-SARS-CoV activity (Boccalone et al., 2020). While the traditional methods of isolation and purification of ginsenoside were challenging and time-consuming, the use of modern-day biotechnological approaches not only enhances its level but also makes the extraction process easier. These approaches include but are not limited to tissue culture, cell suspension culture, protoplast culture, polyploidy, *in vitro* mutagenesis, and hairy root culture (Gantait et al., 2020). For instance, Yu et al. (2016) have reported the enhanced production of ginsenoside by using a fungal strain *Alternaria panax* in cell suspension cultures. The cell wall exudates fungi that contain oligosaccharides and chitin that act as biotic elicitors (Yu et al., 2016).

Jasmonates have been reported to induce oxidative stress and downregulate many genes which lead to an augmentation of ginsenoside in the cell suspension culture. The mechanism behind the role of the elicitor mainly involve the activation of phenylalanine amino lyase which in turn elevates the level of defense compounds and hence ginsenoside biosynthesis (Yu et al., 2002; Kim et al., 2004; Wang et al., 2006).

Mutagenesis and *in vitro* cultures which incorporate genotypic changes in the culture is another popular method used for the enhanced production of ginsenoside. In this method, *P. ginseng* calli are exposed to varying doses of gamma radiation ranging from 10 to 100 Gy (Gray) and for various lengths of time to bring genetic changes and hence to increase the ginsenoside level in callus cultures (Kim et al., 2009, 2013). The increased production of primary ginsenosides was associated with the high expression of squalene epoxidase, dammarenediol synthase, and phytosterol synthase genes (Kim et al., 2013). Summing up, it can be concluded that engineering plant cell and tissue cultures, through *in vitro* mutagenesis, direct gene transfer, and *A. tumefaciens*-mediated transformation could play an important role in the production/enrichment of natural products that are easily repeatable in a short time and thus can be exploited in the fight against COVID-19.

## Commercialization of Plant *In Vitro* Cultures for Secondary Metabolite Production

Advances in biotechnological approaches, particularly plant cell culturing methods, provides new means for commercially valuable, medicinally important plant secondary metabolites (Hussain et al., 2012). Different kinds of strategies have been used, for example, appropriate design of bioreactor systems, optimization of nutrient medium, highly productive line selection, elicitation, two-phase cultivation, and metabolic engineering (Marchev et al., 2020). The scaling-up of *in vitro* plant culturing into large-scale, economically feasible bioreactors provides the sustainable and continuous production of high-valued plant secondary metabolites. Secondary metabolite production in bioreactors depends on pharmacological significance as well as human health benefits. The production of rosmarinic acid and saponins are the selected examples of the commercial production of secondary metabolites *in vitro* (Weathers et al., 2010). Different kinds of bioreactor systems are used to enhance the accumulation of rosmarinic acid such as hairy root cultures or shoot suspension cultures from different plants. Plant suspension culture technology offers a convenient way of upscaling plant *in-vitro* culture systems for the biosynthesis of secondary metabolites. The ease of its upscale is attributed to its shorter cycle of production and simpler methods for bioreactor construction (Marchev et al., 2020). The successful and rapid development of plant metabolic engineering offers an attractive opportunity to increase the content of secondary metabolites in cell and hairy root cultures from aromatic and medicinal plants at a feasible level. Moreover, plant metabolic engineering also makes it possible to understand the molecular biology of the biosynthesis of the secondary metabolites (Pavlov and Bley, 2018). Ginsenoside and taxole, examples of successfully commercialized plant cell suspension cultures, derived specialized metabolites. As reported earlier (Table 1), ginsenosides have proven to be effective against SARS-CoV in inhibiting glycoprotein activity (Wu et al., 2004). Although due to the limited understanding of the molecular basis of plant secondary metabolite biosynthesis, the widespread utilization of the plant suspension cultures platform has yet to be primarily realized (Arya et al., 2020). However, with much research effort, many secondary metabolites achieved a semi-commercial status (Weathers et al., 2010).

Regarding commercialization, the most promising fact associated with plant tissue cultures is that they offer an avenue for the production of these medicinally important phytochemicals in an appropriate bioreactor. The production of the plant *in vitro* cultures is indeed an important prerequisite for the large-scale yield and commercialization of phytomedicine. This is linked with the fact that once carefully selected and optimized, *in vitro* cultures of plants could yield ten times higher phytomedicine than plants grown naturally. However, the production of phytomedicine in bioreactors requires the selection of suitable cell lines/cultures, optimization of culture conditions, application of proper elicitation strategy, immobilization of cells, and efficient downstream processing (Marchev et al., 2020).

Commercial production of secondary metabolites largely depends either on higher market value or high demand which will undoubtedly be high if any of the phytochemicals tested proved effective against the current SARS-CoV virus. The continuous efforts in this field will lead to the controllable and successful production of specific, valuable, and yet unknown plant secondary metabolites (Hussain et al., 2012) against human viruses specifically coronaviruses. Such understanding will ultimately lead to the production of important phytochemicals that are active against SARS-CoV-2.

## Conclusion

Plant biotechnology is a promising platform for exploring the unlimited potential of many diverse medicinal plants. Unfortunately, pandemics like COVID-19 are likely to occur again on a smaller or larger scale due to the array of known and unknown pathogens out there. Plant biotechnology tools and methods such as *in vitro* culture technology is an asset at our disposal to harness the diversity of secondary metabolites produced by different plants. *In vitro* culture technologies can potentially grow any plant anywhere and offer the added value of overproduction of plant cultures, enhanced production of secondary metabolites, and the generation of novel medicinal compounds.

## Author Contributions

TK and MK conceived the idea. TK, KK, MK, NU, and AN each drafted a different section of the manuscript. MK critically reviewed the manuscript. KK and Z-U-RM gathered the data and prepared and formatted the tables. TK, KK, and MK performed the revisions. TK constructed the figures and formatted the manuscript. All authors contributed to the article and approved the submitted version.

## Conflict of Interest

The authors declare that the research was conducted in the absence of any commercial or financial relationships that could be construed as a potential conflict of interest.
